# Mechanisms and roles of membrane-anchored ATG8s

**DOI:** 10.3389/fcell.2025.1532050

**Published:** 2025-01-28

**Authors:** Soo-Kyeong Lee, Sang-Won Park, Deok-Jin Jang, Jin-A. Lee

**Affiliations:** ^1^ Department of Biological Sciences and Biotechnology, College of Life Sciences and Nanotechnology, Hannam University, Daejeon, Republic of Korea; ^2^ Research Institute of Invertebrate Vector, Kyungpook National University, Sangju, Republic of Korea; ^3^ Department of Ecological Science, College of Ecology and Environment, Kyungpook National University, Sangju, Republic of Korea

**Keywords:** autophagy, LC3/GABARAP, LIR motif, non-canonical autophagy, Lando, LAP, probe, deconjugase

## Abstract

Autophagy-related protein 8 (ATG8) family proteins, including LC3 and GABARAP subfamilies, are pivotal in canonical autophagy, driving autophagosome formation, cargo selection, and lysosomal fusion. However, recent studies have identified non-canonical roles for lipidated ATG8 in processes such as LC3-associated phagocytosis (LAP), LC3-associated endocytosis (LANDO), and lipidated ATG8-mediated secretory autophagy. These pathways expand ATG8’s functional repertoire in immune regulation, membrane repair, and pathogen clearance, as ATG8 becomes conjugated to single-membrane structures (e.g., phagosomes and lysosomes). This review examines the molecular mechanisms of ATG8 lipidation, focusing on its selective conjugation to phosphatidylethanolamine (PE) in autophagy and phosphatidylserine (PS) in CASM. We highlight LIR-based probes and LC3/GABARAP-specific deconjugases as critical tools that allow precise tracking and manipulation of ATG8 in autophagic and non-autophagic contexts. These advancements hold therapeutic promise for treating autophagy-related diseases, including cancer and neurodegenerative disorders, by targeting ATG8-driven pathways that maintain cellular homeostasis.

## Introduction

The ATG8-family proteins, which include the Microtubule-associated Protein 1 Light Chain 3 (MAP1LC3) and Gamma-Aminobutyric Acid Receptor-Associated Protein (GABARAP) subfamilies, have evolved in mammals to include seven members: LC3A, LC3B, LC3B2, LC3C, GABARAP, GABARAPL1, and GABARAPL2 (Lane et al., 2013; Shpilka et al., 2011). These proteins share a structural similarity to ubiquitin, including hydrophobic pockets that serve as docking sites for the LC3 interacting region (LIR) of various autophagy-related proteins (Johansen and Lamark, 2020). Additionally, a newly discovered ubiquitin-interacting motif (UIM) contributes to the recruitment of autophagy-related proteins (Marshall et al., 2019).

In the macroautophagy process, ATG8s are synthesized as proproteins and cleaved by ATG4 proteases, exposing a C-terminal glycine. This step is crucial for conjugating ATG8 proteins to the lipid phosphatidylethanolamine (PE) on the autophagosomal membrane, facilitated by two ubiquitin-like conjugation systems (Nieto-Torres et al., 2023; Ichimura et al., 2004; Yu et al., 2012). ATG7 and ATG10 catalyze the ATG5-ATG12 conjugation, which recruits ATG16L1 to form a complex with E3-like activity. This complex mediates ATG8 lipidation (Atg8ylation) through ATG3 and ATG7 (Mizushima, 2020). Lipidated ATG8, in its conjugated form with PE on autophagosomal membranes, plays a pivotal role in the canonical autophagy pathway (Lee and Lee, 2016; Schaaf et al., 2016). It is essential for autophagosome formation, cargo recognition, and the eventual fusion of autophagosomes with lysosomes (Weidberg et al., 2010; Stolz et al., 2014). The lipidation of ATG8 facilitates autophagy induction, membrane elongation and closure, ensuring efficient sequestration and degradation of damaged organelles, misfolded proteins, and other cellular debris (Nieto-Torres et al., 2023; Lee and Lee, 2016). As such, lipidated ATG8 serves as a key marker of autophagosomes and is widely used as a biomarker for autophagy (Mizushima and Komatsu, 2011). While ATG8 proteins primarily perform their functions when lipidated on autophagosomal membranes, some partners interact with non-lipidated ATG8s (e.g., LC3-I) (Reid et al., 2022). Notably, during the autophagy induction stage, GABARAPs play a central role in activating the ULK1 complex (Birgisdottir et al., 2019; Grunwald et al., 2020; Joachim et al., 2015). In addition to autophagy, non-lipidated ATG8 proteins have been implicated in cellular repair mechanisms and vesicular trafficking. These roles highlight their involvement in non-autophagic processes, such as regulating cellular signaling and maintaining homeostasis (Schaaf et al., 2016). This broadens the functional spectrum of ATG8, extending beyond its classical autophagic role to include novel mechanisms critical for cellular integrity.

Non-canonical autophagy further diversifies the roles of ATG8-family proteins. In addition to their well-established functions in canonical pathways, non-canonical autophagy introduces unique mechanisms involving single-membrane structures. Unlike the double-membrane autophagosomes in canonical pathways, non-canonical processes involve the conjugation of ATG8 proteins to phosphatidylserine (PS) instead of PE. Atg8ylation refers to the attachment of ATG8-family proteins to cellular membranes, specifically phospholipids like PE and PS. This process mimics ubiquitination and relies on an enzymatic cascade involving E1, E2, and E3-like enzymes. Atg8ylation plays a crucial role in recognizing and remodeling cellular membranes under various stress conditions and regulating their turnover (Deretic and Lazarou, 2022; Carosi et al., 2021). Furthermore, Atg8ylation on single-membrane compartments supports diverse cellular functions beyond degradation, including secretion, immunity, and membrane remodeling (Reid et al., 2022; Kumar and Mills, 2023; Fletcher et al., 2018; Takáts et al., 2018; Wang et al., 2024). For instance, LC3-associated phagocytosis (LAP) enables the degradation of extracellular particles, such as apoptotic cells and pathogens, by conjugating ATG8 proteins to single-membrane phagosomes (Reid et al., 2022). Similarly, Conjugation of ATG8 to Single Membranes (CASM) represents an alternative lipidation process that is crucial for maintaining lysosomal function and regulating immune responses (Durgan et al., 2021). Together, these processes underscore the versatility of ATG8 functions, highlighting their critical roles in both autophagic and non-autophagic pathways.

Given the complexities of these processes, advanced molecular tools are essential for understanding the regulatory mechanisms of both lipidated and non-lipidated ATG8. The development of LIR-based probes, which specifically detect ATG8-LIR interactions, provides unprecedented precision in tracking ATG8 engagement with its binding partners in both autophagic and non-autophagic contexts. Additionally, LC3/GABARAP deconjugases, which selectively remove ATG8 from membranes, are key tools that allow researchers to dissect the dynamic balance of ATG8 lipidation and delipidation, revealing its broader roles in cellular homeostasis and disease pathways.

This review will delve into the multifaceted roles of membrane-anchored ATG8 proteins, emphasizing the importance of tools like LIR-based probes and deconjugases in studying the functional dynamics of ATG8 in autophagy and other processes. By shedding light on these processes, we aim to highlight the therapeutic potential of modulating ATG8 functions in the context of disease.

## ATG8-family proteins: essential roles in autophagosome formation, cargo selection, and lysosomal fusion

**FIGURE 1:**
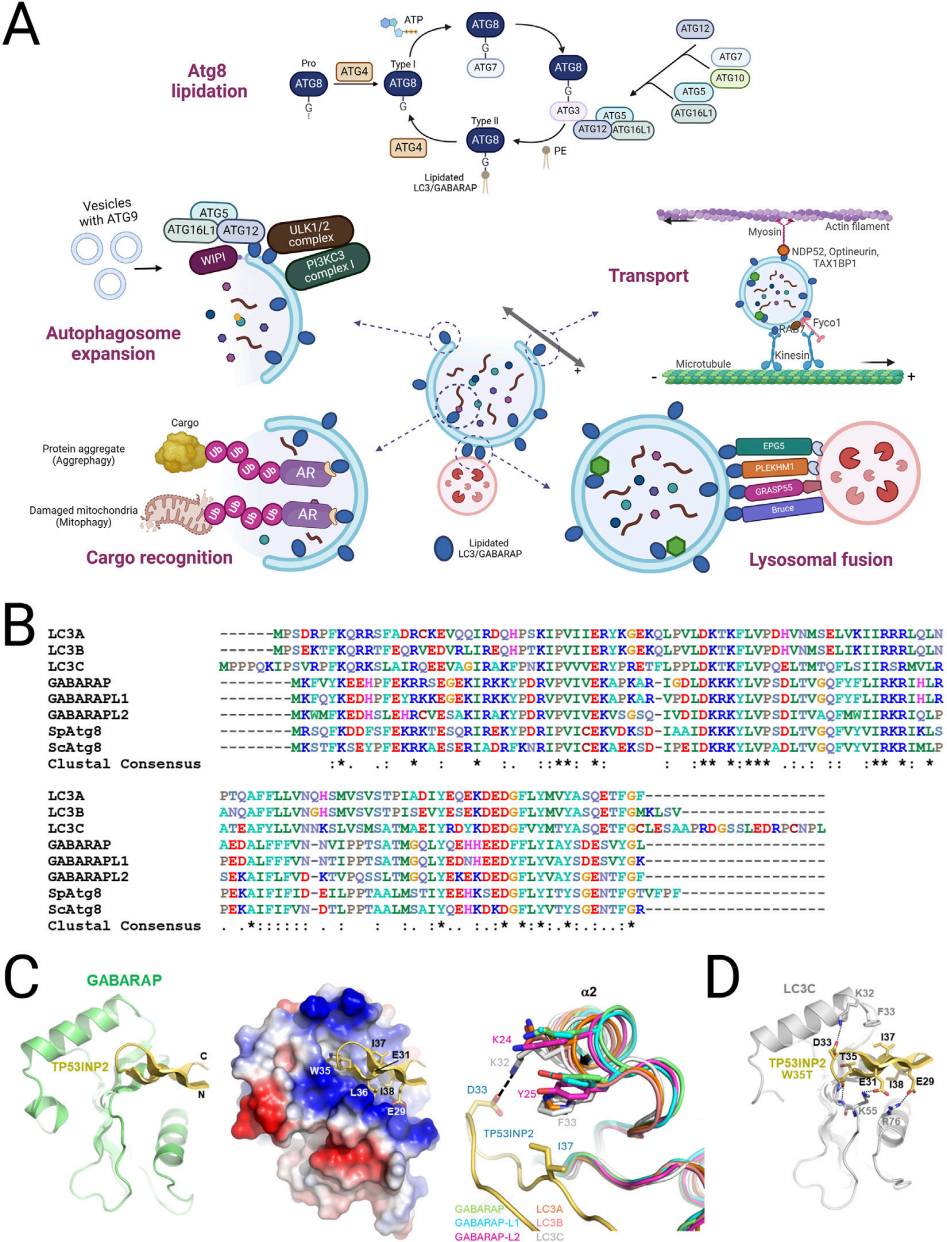
Function, classification and structure of mATG8 family proteins in autophagy. **(A)** A schematic illustration of the mATG8 lipidation process and the functions of lipidated mATG8 proteins in autophagosomal membranes. Pro-mATG8 proteins are cleaved by ATG4 enzymes to form cytosolic mATG8 (Type I), which is then conjugated to phosphatidylethanolamine (PE) through a series of enzyme actions: E1 (ATG7), E2 (ATG3), and E3 (the ATG5–ATG12–ATG16L1 complex), converting it into lipidated mATG8 (Type II). Lipidated LC3/GABARAP proteins perform multiple functions, including autophagosome expansion, transport, cargo recognition, and lysosomal fusion, by providing accessible sites on the autophagosomal membrane for autophagy machinery proteins. They bind to LIR-containing receptors on cargo destined for degradation. **(B)** Sequence alignment of LC3/GABARAP and fungal Atg8 proteins. **(C)** The crystal structure of the LIR (TP)-GABARAP fusion was prepared by combining GABARAP and LIR from different fusion molecules interacting within the crystal. The structure displays a ribbon model on the left and electrostatic surface potentials with a ribbon model of LIR peptides in the center, highlighting interacting LIR residues. On the right, a structural comparison of the α2 helix across LC3/GABARAP proteins is shown, using Cα atoms for superimposition. The following PDB IDs were used: LC3A, 5CX3; LC3B, 5D94; LC3C, 3VVW; GABARAP, 1GNU; GABARAP-L1, 6HOI; and GABARAP-L2, 4CO7. **(D)** A modeled structure of the LIR [TP(T)]-LC3C complex is also shown, with potential electrostatic or hydrogen-bond interactions represented by a stick model.

**FIGURE 2:**
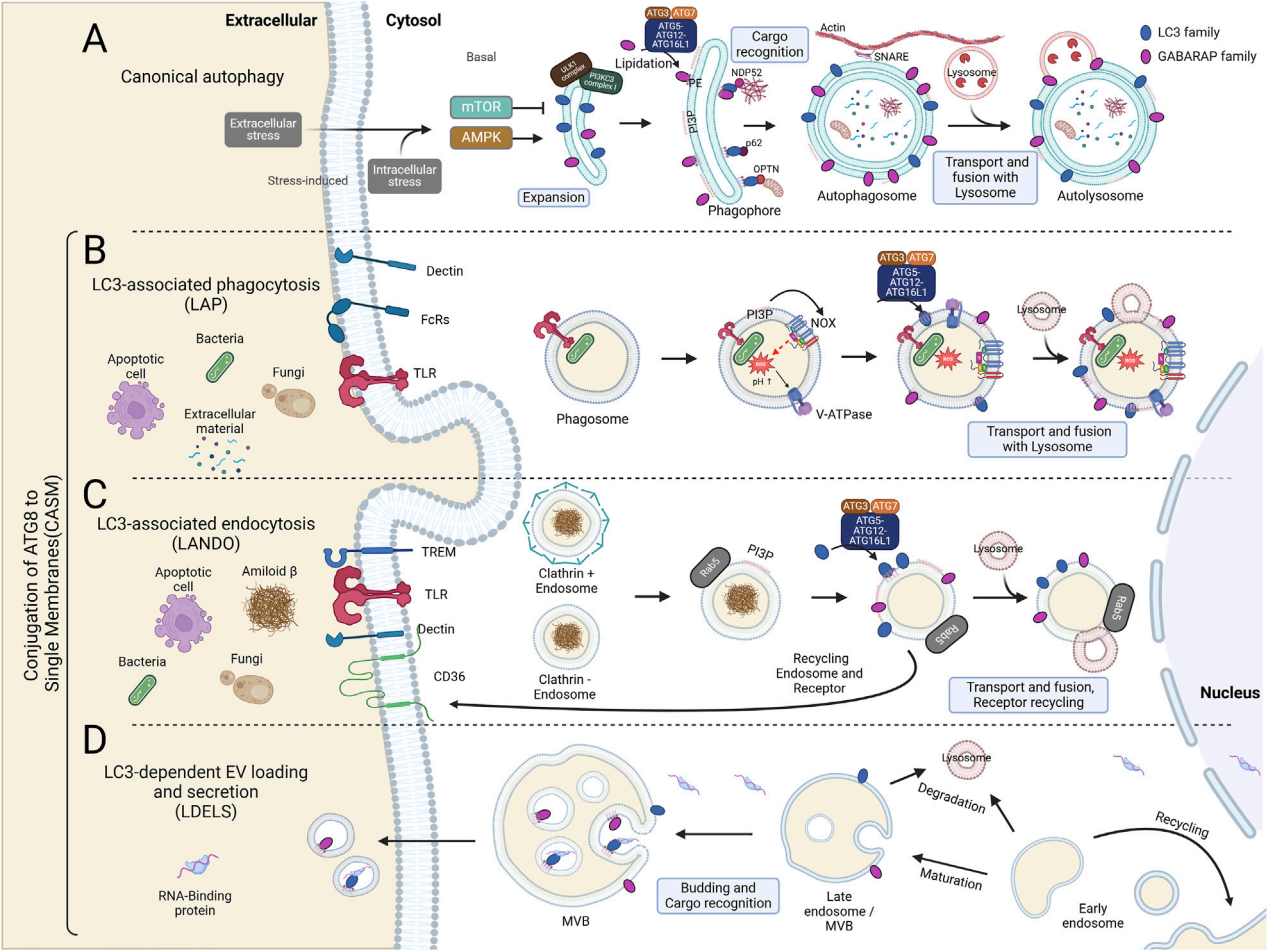
Canonical and Non-Canonical Autophagy Pathways: Canonical Autophagy and CASM. **(A)** Autophagy initiation requires the ULK1 kinase complex, regulated by AMPK and mTOR as activator and inhibitor, respectively. During the expansion phase, AMPK phosphorylates ULK1, which includes FIP200, ATG13, and ATG101, thereby activating the class III PI3K complex composed of BECN1, AMBRA1, ATG14L, VPS15, and VPS34. This complex generates phosphatidylinositol 3-phosphate (PI3P), promoting phagophore elongation. Within the phagophore, mATG8 proteins recruit cargo for degradation. mATG8 is cleaved by ATG4 and lipidated by the combined actions of ATG7, ATG3, and the ATG5-12-16L1 complex, anchoring it to autophagosomal membranes. The mature, closed autophagosomes are transported via SNARE proteins to actin filaments and ultimately fuse with lysosomes through mATG8s, enabling degradation. **(B)** LC3-Associated Phagocytosis (LAP) process is initiated in murine macrophages through interactions between extracellular components and receptors, including Dectin (a phagocytic receptor), Fc receptors (FcRs), and Toll-like receptors (TLRs). Unlike canonical autophagy, LAP formation does not require the ULK1 complex. Upon phagocytosis, PI3P is generated by the VPS34 complex on the single phagosomal membrane, activating the NADPH oxidase complex (NOX). NOX activation leads to ROS production within the phagosomal lumen, raising pH levels. This high pH then promotes the assembly of V-ATPase on the phagosomal membrane. V-ATPase interacts with the WD40 domain of ATG16L1, subsequently recruiting the ATG5-ATG12-ATG16L1 complex, which facilitates the conjugation of LC3 or GABARAP to phosphatidylserine (PS) and phosphatidylethanolamine (PE) on the phagosomal membrane. The mature mATG8-conjugated phagosome is ultimately degraded through lysosomal fusion. **(C)** LC3-Associated Endocytosis (LANDO) is linked with recycling membrane receptors, such as TLRs, TREM, and CD36, and plays a role in regulating inflammation within the immune system. Similar to LAP, LANDO does not require the ULK1 complex. Cytosolic endosomes marked by Rab5 interact with the VPS34 complex to generate PI3P on the single-membrane endosome. PI3P enables the conjugation of mATG8 proteins, such as LC3 and GABARAP, through the ATG5-ATG12-ATG16L1 complex. This process facilitates endosome fusion with lysosomes for degradation or recycling via the ATG8-mediated endosomal pathway. **(D)** LC3-Dependent EV Loading and Secretion (LDELS) is a regulated pathway for degrading proteins, RNAs, and lipids through extracellular vesicles (EVs). EVs, derived from multivesicular bodies (MVBs), carry cargos recognized by mATG8 proteins, which also facilitate the secretion of LC3-dependent EVs.

The ATG8-family proteins, comprising LC3 and GABARAP subfamilies, are indispensable for the proper execution of autophagy, particularly in the stages of autophagosome formation, cargo selection, and lysosomal fusion. These proteins undergo lipidation, a key modification wherein they conjugate to the lipid PE, facilitating their anchoring to autophagosomal membranes. This lipidation is essential for the elongation and closure of the autophagosome, a double-membrane vesicle that sequesters cellular material for degradation (Figure 1; Figure 2A).

### Autophagosome formation

During autophagy, the initiation of autophagosome formation begins with the assembly of a phagophore. Lipidated LC3 and GABARAP proteins are rapidly recruited to the expanding phagophore, where they help stabilize the growing membrane and promote its expansion. The association of ATG8-family proteins with the autophagosomal membrane is critical for membrane curvature and vesicle completion, allowing the autophagosome to engulf intracellular cargo, including damaged organelles and misfolded proteins (Alemu et al., 2012). The lipidation of LC3 to the autophagosomal membrane has become a hallmark of autophagy induction and is commonly used as a marker in autophagy studies. Intriguingly, human cells lacking all ATG8 proteins exhibit inefficient autophagosome formation and impaired fusion of autophagosomes with lysosomes. This defect can be rescued by GABARAP family proteins but not by LC3 family proteins (Vaites et al., 2017; Nguyen et al., 2016). Thus, GABARAP proteins (GABARAP, GABARAPL1, GABARAPL2) seem to be more active in the later stages of autophagosome formation, contributing significantly to phagophore elongation and the maturation of autophagosomes. GABARAP proteins are particularly involved in stabilizing the interaction between autophagy-related proteins, such as the ATG12-ATG5 complex, facilitating efficient membrane expansion (Weidberg et al., 2010). The efficient lipidation and recruitment of ATG8 proteins are regulated by the ATG12-ATG5-ATG16L1 complex, which facilitates the conjugation process at the membrane, ensuring that the phagophore elongates and eventually forms a closed autophagosome (Figure 2A) (Corkery et al., 2023).

### Cargo selection

ATG8 proteins are also integral in selective autophagy processes, acting as molecular bridges between the autophagosome and specific substrates to be degraded. LC3 and GABARAP interact with cargo adaptors such as p62/SQSTM1, NDP52, and OPTN, which recognize ubiquitinated proteins and other cargo earmarked for degradation. The presence of LC3 interacting regions (LIRs) or GABARAP-interacting motifs (GIM) in these adaptors allows for a direct binding to ATG8-family proteins, ensuring the efficient loading of substrates into the autophagosome. While LC3 is involved in the early stages of cargo recognition and loading, GABARAP ensures the autophagosome’s maturation and eventual fusion with lysosomes for cargo degradation (Weidberg et al., 2010; Birgisdottir et al., 2019; Pankiv et al., 2007). This process is critical for maintaining cellular homeostasis, especially in contexts of stress where damaged proteins and organelles must be efficiently degraded.

### Lysosomal fusion

Once the autophagosome is fully formed, ATG8 proteins are essential for the subsequent fusion of the autophagosome with lysosomes. This fusion is crucial for the degradation of autophagic cargo, as lysosomal hydrolases break down the engulfed material. GABARAP subfamily proteins, in particular, play a prominent role in this fusion process by interacting with proteins involved in vesicle trafficking, such as the SNARE complex, like syntaxin-17, which mediates the docking and fusion of the autophagosome with the lysosome. The removal of ATG8 from the autophagosomal membrane by ATG4 proteases after lysosomal fusion allows for the recycling of ATG8 proteins, ensuring their availability for future rounds of autophagy.

In summary, while both LC3 and GABARAP proteins share overlapping roles in autophagosome formation, they operate at different stages, with LC3 contributing more significantly to early phagophore expansion and cargo recognition and GABARAP participating in the elongation and eventual fusion of the autophagosome with lysosomes (Weidberg et al., 2010; Wang et al., 2016; Florey et al., 2015; Kabeya et al., 2000). However, recent studies have also shown that the ATG conjugation system is required for the degradation of the inner membrane of autophagosomes and the trafficking of autophagosomes (Tsuboyama et al., 2016). These processes are crucial for maintaining cellular homeostasis, particularly under stress conditions, and highlight the critical roles of LC3 and GABARAP in both selective and non-selective autophagy pathways.

## ATG8 proteins in non-canonical processes

In addition to their roles in canonical autophagy, lipidated LC3 and GABARAP are central to various non-canonical autophagy pathways (CASM). These pathways, in contrast to canonical autophagy, involve the conjugation of ATG8 proteins to single-membrane structures, such as phagosomes and lysosomes, rather than the double membranes of autophagosomes. This non-canonical lipidation process plays a critical role in several cellular functions, including immune regulation, membrane repair, and pathogen clearance, thereby expanding the functional repertoire of ATG8-family proteins (Figure 2).

### CASM

CASM represents a critical deviation from canonical autophagy, specifically in how ATG8 proteins like LC3 are conjugated to single-membrane structures such as lysosomes and endosomes, rather than double-membrane autophagosomes. Its discovery and subsequent research have broadened our understanding of non-canonical autophagy and opened up new avenues of study, particularly concerning the molecular machinery involved and its physiological significance. CASM was first described in relation to the recruitment of ATG8-family proteins to single membranes under certain stress conditions, independent of the canonical autophagy initiation complex. Florey et al. (2015) provided early insights into this process by showing that lysosomotropic agents such as chloroquine (CQ) could recruit LC3 to single-membrane lysosomes, a process distinct from autophagosome formation. This observation led to the identification of a novel ATG8 lipidation system, later termed CASM. Recent studies by Durgan and Florey (2022) have expanded upon this finding by revealing the critical role of the ATG16L1-WD40 domain and its interaction with V-ATPase on lysosomal membranes as a central component of CASM (Figures 2B, C) (Takáts et al., 2018; Alemu et al., 2012; Vaites et al., 2017).

In canonical autophagy, ATG8-family proteins (such as LC3 and GABARAP) are conjugated to PE on the double membranes of autophagosomes. This process requires an autophagy initiation complex composed of ULK1, FIP200, ATG13, and ATG101, which orchestrates the early steps of autophagosome formation. This lipidation is essential for autophagosome elongation, cargo recognition, and eventual fusion with lysosomes. This process includes a well-characterized enzymatic cascade involving ATG7 (E1-like), ATG3 (E2-like), and the ATG12–ATG5-ATG16L1 complex, which acts similarly to an E3 ligase, facilitating the conjugation of ATG8 to PE. Once lipidated, these proteins promote the selective or non-selective sequestration of cellular components into the autophagosome for degradation (Lane et al., 2013; Shpilka et al., 2011). CASM is a process where ATG8-family proteins are conjugated to PE or PS (Durgan et al., 2021), and occurs on single-membrane structures, such as endolysosomal membranes (Florey et al., 2015; Jacquin et al., 2017), Golgi compartments (Gao et al., 2016; Liu et al., 2023), or phagosomes during LAP (Durgan and Florey, 2022). The molecular mechanism underlying CASM involves several key steps. CASM operates independently of the canonical autophagy initiation complex (ULK1, FIP200, ATG13, ATG101). First, under stress conditions such as lysosomal damage or pathogen invasion, V-ATPase recruits ATG16L1 to the lysosomal membrane. This interaction is specific to the WD40 domain of ATG16L1, which is not required for canonical autophagy. Once ATG16L1 is localized to the lysosomal membrane, it promotes the conjugation of ATG8 proteins, such as LC3, to PS through the action of the ATG8 lipidation machinery, composed of ATG7 (E1-like enzyme), ATG3 (E2-like enzyme), and the ATG12-ATG5-ATG16L1 complex (Corkery et al., 2023; Florey et al., 2015; Florey et al., 2011). During CASM, ATG16L1’s WD40 domain plays a key role by interacting with vacuolar H (+)-ATPase (V-ATPase), recruiting the ATG8 conjugation system directly to lysosomal membranes (Florey et al., 2015; Florey et al., 2011). More recently, we have demonstrated a direct interaction between the V1H subunit of the V-ATPase and ATG16L1 (Timimi et al., 2024).

A recently described ATG16L1-independent form of CASM involves TECPR1 forming a complex with ATG5–ATG12, inducing ATG8 lipidation on single-membrane structures (Corkery et al., 2023; Figueras-Novoa et al., 2024). This pathway depends on sphingomyelin exposure in damaged membranes and has been proposed as sphingomyelin–TECPR1-induced LC3 lipidation (STIL) (Corkery et al., 2023; Boyle et al., 2023). In STIL, LC3 lipidation plays a critical role in marking damaged membranes, facilitating their repair or removal, and serving as a key cellular defense mechanism against membrane stress or pathogen-induced damage.

CASM, through the membrane conjugation of GABARAP, is required for TFEB activation downstream of TRPML1 to maintain lysosomal homeostasis (Goodwin et al., 2021). Recent studies have demonstrated that CASM plays a broader role in immune regulation, particularly in the context of pathogen clearance and lysosomal maintenance. CASM plays a critical role in modulating inflammatory responses. For instance, Xu et al. (2019) showed that CASM contributes to lysosomal stability, preventing excessive lysosomal permeabilization that could lead to uncontrolled inflammatory signaling (Durgan and Florey, 2022). Furthermore, Ulferts et al. (2021) highlighted the involvement of CASM in autoimmune disorders, where dysregulation of ATG8 conjugation to lysosomal membranes exacerbates inflammatory pathways, suggesting a protective role of CASM in maintaining immune homeostasis (Florey et al., 2015; Gao et al., 2016; Timimi et al., 2024). These findings collectively underscore the broader role of CASM in immune regulation, highlighting its importance in pathogen clearance, inflammation control, and the prevention of autoimmune responses.

However, the precise molecular mechanisms through which CASM influences these immune processes remain to be fully elucidated, necessitating further research to uncover the intricate details of ATG8-family protein interactions in immune contexts.

### LAP

In LAP, ATG8 proteins are recruited to single-membrane phagosomes during the process of phagocytosis, where cells engulf pathogens, apoptotic cells, or debris. LAP differs from canonical autophagy by bypassing autophagosome formation and instead conjugating LC3 to PE on the phagosomal membrane. This conjugation is essential for phagosome maturation, promoting their fusion with lysosomes and subsequent degradation of the engulfed material (Jenzer et al., 2019). It operates without the autophagy initiation complex (ULK1, FIP200, ATG13, ATG101), yet still utilizes shared components such as ATG7, ATG3, and the ATG12-ATG5-ATG16L1 complex for membrane lipidation in a different context. Interestingly, a recent study has revealed that all ATG8 proteins can alternatively conjugate to PS rather than PE during LAP or in the context of influenza virus infection (Durgan et al., 2021). LAP plays a key role in immune regulation by ensuring the efficient clearance of apoptotic cells and pathogens, preventing chronic inflammation and autoimmune disorders. For example, LAP-mediated phagocytosis of dead cells and pathogens suppresses excessive immune responses, thereby maintaining immune homeostasis. The process is also critical for pathogen clearance, as it helps degrade invasive microorganisms that escape canonical autophagic degradation pathways. Indeed, the loss of functional lipidation machinery is associated with the failure of LAP, which is linked to inflammation, autoimmune disorders, trafficking, and secretion (Figure 2B) (Heckmann et al., 2017).

### LANDO

The ATG8 conjugation machinery also appears to be crucial in a mechanistically distinct process called LC3-associated endocytosis (LANDO). LC3 is recruited to single-membrane entotic vacuoles, macropinosomes, and apoptotic cell-containing phagosomes via lipidation machinery, independent of autophagosome formation. This suggests that autophagy proteins target single-membrane vacuoles in the absence of pathogens, mediating lysosome fusion and degradation of internalized cells, with potential roles in both tumor suppression and pathogen defense (Florey et al., 2011). In addition, LC3 and GABARAP lipidation at single membranes, particularly in late endosomes, plays a role in the selective degradation of autophagy receptors during starvation. This process occurs independently of canonical autophagy, utilizing endosomal microautophagy and the ESCRT machinery to rapidly degrade specific proteins (Mejlvang et al., 2018). LANDO has been particularly highlighted for its role in regulating neuroinflammation. It was found to be crucial for maintaining homeostasis in microglia, which are immune cells of the central nervous system. Dysfunction in LANDO is associated with increased neuroinflammation, and impairments in LANDO pathways have been linked to Alzheimer’s disease, suggesting that targeting LANDO could be a therapeutic approach to neurodegenerative disease (Figure 2C) (Heckmann et al., 2019).

### Lipidated ATG8-mediated secretory autophagy

The role of LC3 and GABARAP lipidation at single membranes is highlighted in secretory autophagy, a process where cytosolic cargo, like pro-inflammatory cytokines and protein aggregates, is secreted through non-conventional pathways. Intact extracellular vesicles produced via secretory autophagy containing LC3B are generally small, while exosomes contain CD63 and the ESCRT-I complex subunit (Chen et al., 2019). In secretory autophagy, LC3 and GABARAP are conjugated to single membranes, such as those of autophagosome-like structures or multivesicular bodies (MVBs), facilitating cargo selection and export from the cell. Notably, LC3 lipidation assists in the formation of amphisomes, which are hybrid structures between autophagosomes and MVBs. These structures mediate the secretion of proteins like IL-1β and α-synuclein without conventional lysis. Lipidation of LC3 and GABARAP on single membranes seems to be critical for the fusion of vesicles with the plasma membrane during secretion. This process helps clear intracellular aggregates and contributes to non-lytic microbial egress, supporting pathogen dissemination while sparing the host cell (Figure 2D). Indeed, defects in secretory autophagy have been associated with various human diseases, including Crohn’s disease, asthma, type II diabetes, Alzheimer’s disease, cancer, and bacterial or viral infections (New and Thomas, 2019).

## Lysosomal damage repair and regulation of stress granules

Recent studies have brought attention to the role of ATG8s in the repair of damaged lysosomes (Cross et al., 2023; Jia et al., 2022), beyond previously identified mechanisms such as CASM, LAP, LANDO, and LDELS. For example, lysosomal damage induced by LLoMe (a drug that causes lysosomal damage through reverse peptidase activity by Cathepsin C) under conditions promoting stress granule formation has been shown to recruit SG proteins, such as NUFIP2 and G3BP1, to the damaged lysosome. This interaction is mediated by Atg8ylation, particularly involving the GABARAP family proteins (Jia et al., 2022). While the interaction between GABARAP and NUFIP2 enhances mTOR inhibition at damaged lysosomes, it simultaneously reduces the availability of NUFIP2 required for stress granule formation. These findings suggest that ATG8s, including GABARAP, play a dual role in regulating damaged lysosomes and modulating stress granule formation (Jia et al., 2022).

Other studies have reported that ATG8s play a critical role in microlysophagy for the repair of damaged lysosomal membranes. It has been shown that lysosome-damaging agents, such as LLOMe, monensin, and nigericin, which enhances GABARAP activity and its interaction with damaged lysosomes (Ogura et al., 2023). The non-canonical lipidation of ATG8s, particularly GABARAP, at damaged lysosomal membranes facilitates interactions with ESCRT proteins, such as ALIX, and promotes the assembly of the ESCRT complex, including CHMP family proteins. Subsequently, GABARAP drives lysosomal membrane engulfment through the assembly of the ESCRT complex, thereby contributing to the repair of damaged lysosomal membranes (Ogura et al., 2023).

ATG8 proteins demonstrate remarkable versatility by repairing damaged lysosomal membranes and regulating stress granules through processes such as Atg8ylation on single membranes. These mechanisms highlight their ability to respond to cellular stressors and facilitate immune defense. The conjugation of ATG8 to single membranes, as seen in these pathways, underscores the broader functional scope of ATG8 proteins beyond traditional autophagy. Through their roles in membrane repair, immune modulation, and pathogen clearance, ATG8 proteins act as crucial regulators of cellular homeostasis and immune defense.

However, there is still much to uncover regarding the functional specialization of ATG8 family members in autophagy-related degradative pathways, particularly concerning their roles in non-canonical processes. Research has primarily focused on LC3 proteins in single-membrane degradative vesicles, leaving gaps in our understanding of the contributions of GABARAP proteins. Although early evidence indicates that GABARAPL2 associates with endosomal-origin single-membrane vesicles, both LC3 and GABARAP proteins may play essential roles in these pathways. The precise roles of these proteins, especially how they interact with transport and fusion proteins, remain unclear.

Tools to study lipidated LC3 and GABARAP specifically on single membranes are underdeveloped, making it challenging to fully elucidate their roles in non-canonical pathways. The development of advanced technologies will be crucial for dissecting these functions, and future sections will elaborate on the emerging methods designed to address these challenges.

**FIGURE 3:**
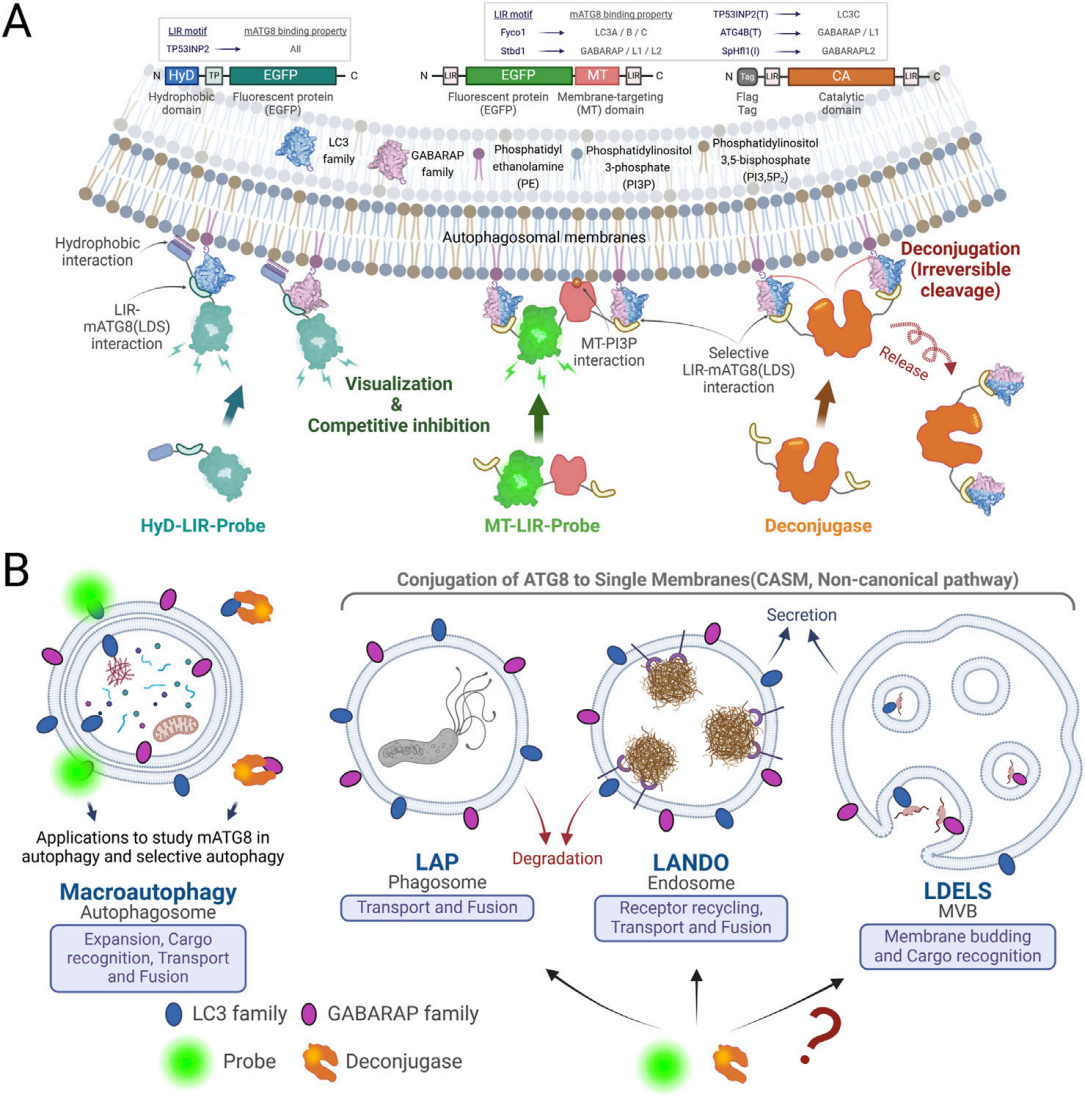
Mechanism of LIR-Based Probes and LC3/GABARAP Deconjugases, and Their Applications in Research and Therapy. **(A)** Schematic diagram of LIR based probes and deconjugases: The HyD-LIR probe illustrated on the left effectively detects mATG8s present on autophagosomal membranes through the synergy of a short hydrophobic domain and LIR. The MT-LIR probe shown in the middle of the picture contains the MT domain of RavZ, which binds PI3P, and selective LIRs on both terminals, allowing it to specifically bind to specific mATG8. The deconjugase on the right consists of the catalytic domain of RavZ and selective LIRs that irreversibly cleave specific mATG8 on autophagosomal membranes. LIR-based probes are commonly used to visualize autophagosomes by weak and transient expression. On the other hand, continuous and overexpression of the probes inhibits autophagy by blocking the binding of mATG8 to other autophagy-related proteins in the autophagosomal membranes. The deconjugases can be expressed to remove specific mATG8 proteins from the autophagosomal membranes to form special situations where LC3s or GABARAPs are lacking, which are different from mATG8 knock-out cells. **(B)** Considerations for the use of LIR-based probes and deconjugases. Probes and deconjugases are used across the entire process of conventional and selective autophagy. The utilisation of probes and deconjugases in the extended functions of mATG8 proteins conjugated to a single membrane (LAP, LANDO and LDELS) is useful for studying the contribution and importance of mATG8 proteins in each pathway, and further enables new studies of mATG8.

Recently, new technologies have been developed to better elucidate the functions of lipidated LC3 and GABARAP. Tools such as LIR-based probes and LC3/GABARAP deconjugases now enable precise monitoring of the conjugation and deconjugation of these proteins, providing critical insights into their regulatory roles in non-canonical autophagy pathways like CASM. In the next section, we will introduce these innovative tools and explore how they are advancing our understanding of ATG8-family protein functions in these pathways (Figure 3).

## LIR-based probes: advancing the detection of ATG8-LIR interactions in autophagy and beyond

The development of LIR-based (Hydrophobic Domain-LC3-Interacting Region) probes has significantly enhanced the study of ATG8-family proteins, particularly their interactions with LIR in autophagic and non-autophagic processes. These probes allow for the precise detection of ATG8-LIR interactions, which are central to understanding how ATG8 proteins facilitate selective autophagy, cargo recognition, and their broader roles in non-canonical processes like LAP and membrane repair (14,24).

LIR-based probes are designed to specifically bind to ATG8-family proteins, including LC3 and GABARAP, by recognizing the hydrophobic pockets that mediate interactions with LIRs or GIM found in various autophagy cargo receptors.

LIR and GIM were identified as critical elements in selective autophagy, allowing specific proteins to bind to LC3 or GABARAP family proteins. These motifs were discovered as sequences that interact with distinct hydrophobic pockets on LC3/GABARAP proteins, aiding in the recruitment of autophagy-related cargo receptors and adaptors (Table 1). The LIR, typically characterized by the consensus sequence [W/F/Y]-X-X-[L/I/V], was first identified in studies such as that by Noda et al., which demonstrated how autophagic proteins utilize this motif to bind LC3 proteins and drive autophagy (Noda et al., 2008). This initial discovery highlighted how various autophagy receptors use LIRs to interact selectively with LC3, promoting the autophagic process through a common binding mechanism. The GABARAP Interaction Motif (GIM), with the core sequence [W/F]-[V/I]-X2-V, was later identified as a variation of the LIR that demonstrates higher selectivity for GABARAP proteins over LC3 proteins. This selective interaction was elucidated in a study by Rogov et al., which revealed that PLEKHM1 and other adaptor proteins use GIMs to selectively bind GABARAPs (Rogov et al., 2017). This specificity was shown to be crucial for the unique roles of GABARAP proteins in autophagy, differentiating them from LC3 proteins in certain cellular contexts.

**TABLE 1:** Summary of the normalized binding property of GIM and LIR motifs.

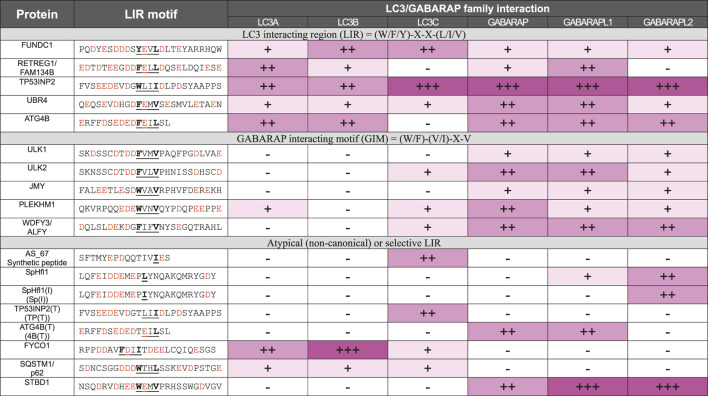

This table summarizes the normalized binding properties of GIM and LIR motifs with various LC3 and GABARAP proteins. Binding strength is categorized as follows: —: no interaction; +: Weak interaction; ++: Moderate interaction; +++: Strong interaction. Normalized A:C ratio values are expressed as mean ± standard deviation. Core regions in GIM and LIR motifs are highlighted in bold. This table reuses data from my previously published study [Sang-Won Patk et al., Autophagy (2022)] (https://doi.org/10.1080/15548627.2022.2132040), adapted with permission.

In general, consensus-sequence LIRs generally exhibit stronger binding to multiple LC3/GABARAP proteins than non-canonical LIRs. Previous structural studies have elucidated the atomic-level mechanisms of atypical LIR interactions with mATG8 proteins. The LIR (TP) conformation shows canonical binding, where residues W35 and I38 bind to the W-site and L-site, respectively, with only E29 forming an electrostatic interaction with GABARAP (R67), indicating that binding affinity largely depends on the core LIR structure. Similar to CALCOCO2’s non-canonical core sequence (I133-L-V-V136), LIR [TP (T)] forms specific interactions with LC3C: I37 and D33 establish hydrophobic and electrostatic interactions with F33 and K32, while E29 and E31 form electrostatic interactions with LC3C’s R76 and K55. The conservation of K55 and R76 in all mATG8 proteins, along with the unique presence of F33 in LC3C, suggests F33’s role in LC3C’s binding specificity. Additionally, atypical LIR variants, such as 4B [T] with a T to F substitution in ATG4B’s LIR motif, selectively bind to GABARAP and GABARAPL1, while the atypical LIR (Sp) of SpHfl1 and Sp [I] specifically target GABARAPL2 (Table 1) (Park et al., 2023).

## LIR-based probes as sensors and inhibitors of ATG8 interactions

Hydrophobic (HyD)-LIR probe: Previous research showed that Aplysia phosphodiesterase 4 (ApPDE4) short form N-terminal moderate HyD was insufficient to mediate membrane association. However, the N-terminal moderate HyD of ApPDE4 could be stably localized to the plasma membrane when combined with a flanking basic-rich domain capable of electrostatic interaction with acidic phospholipids (Kim et al., 2014). HyD, the N-terminal 20 amino acids of the short form of ApPDE4, can be combined with LIRs to effectively detect mATG8 proteins on autophagic membranes (Lee et al., 2017).

Membrane targeting (MT)-LIR probe: The MT domain of the RavZ protein binds to PI3P, a major constituent lipid of autophagosomes (Horenkamp et al., 2015). The MT domain alone is not sufficient for binding to localize to the membrane, but it works synergistically with the α3 helix in the catalytic domain of RavZ or the LIRs to strongly target the membrane (Park et al., 2019a). Thus, MT-LIR probe fusing MT domain with LIRs that selectively bind to some of the LC3/GABARAP proteins effectively detect specific mATG8 in autophagosomes (Park et al., 2023; Park et al., 2019b).

LIR-based probes, such as HyD-LIR or MT-LIR probes, offer a powerful tool for studying ATG8-family protein interactions by targeting LIRs. These LIR-based probes underline the potential of such engineered probes in advancing autophagic membrane studies, offering highly selective tools for the detection and investigation of mATG8 proteins and other autophagic markers (Figure 3A).

These probes have the unique ability to act as sensors or inhibitors depending on their expression levels and modifications. At lower concentrations, LIR-based probes can serve as sensors by detecting specific ATG8-LIR interactions and lipidated ATG8 on autophagosomes or membranes (Park et al., 2023; Park et al., 2019b; Jang et al., 2024), allowing researchers to map and quantify ATG8 engagement with cargo receptors like p62/SQSTM1, NDP52, or OPTN in both autophagy and non-canonical processes such as LAP.

On the other hand, at higher expression levels, LIR-based probes can competitively bind to ATG8-family proteins, acting as inhibitors of LIR-mediated interactions. By saturating the available LIR binding sites on ATG8 proteins, these probes can block endogenous LIR-containing proteins from interacting with ATG8, thereby inhibiting selective autophagy pathways or non-canonical functions like CASM. This makes LIR-based probes a valuable tool for not only detecting ATG8 interactions but also manipulating these interactions to study their functional consequences under various physiological or pathological conditions. The ability to tune the activity of LIR-based probes by modulating their expression or concentration opens new avenues for their use in both basic research and therapeutic applications. As sensors, they provide real-time insights into ATG8-dependent processes, while as inhibitors, they offer a targeted approach for disrupting autophagy-related pathways in diseases such as cancer and neurodegeneration.

The ability to detect ATG8-LIR interactions with precision has profound implications for the study of autophagy-related diseases. Alzheimer’s disease (AD) and Parkinson’s disease (PD) are neurodegenerative disorders characterized by abnormal protein accumulation, leading to cognitive and motor impairments, respectively. In AD, amyloid β and tau tangles build up due to an imbalance in production and clearance, with autophagy playing a critical role in their degradation (Heckmann et al., 2019; Nalivaeva et al., 2012; Nixon et al., 2005). Similarly, in PD, α-synuclein accumulates in Lewy bodies, and autophagy, alongside other degradation pathways, is essential for its clearance. Genetic factors and proteins like presenilin in AD and LRRK2 in PD highlight the importance of autophagic pathways in managing these protein aggregates (Wakabayashi et al., 2013; Ebrahimi-Fakhari et al., 2011; Wong and Cuervo, 2010), underscoring autophagy as a potential therapeutic target in both diseases.

Given these insights, LIR-based probes offer promising applications as both sensors and inhibitors in the context of neurodegenerative diseases, specifically in the modulation of autophagic pathways associated with amyloid β and α-synuclein degradation in AD and PD, respectively. LIRs have a selective binding affinity for LC3 and GABARAP proteins on autophagic membranes, making LIR-based probes valuable as real-time sensors for tracking autophagic flux in both disease models. As sensors, LIR-based probes could monitor disruptions in autophagic degradation within neurons and glial cells, facilitating the study of amyloid β clearance in AD and Lewy body clearance in PD.

In addition, LIR-based probes can serve as inhibitors by binding directly to autophagy-related proteins such as LC3 or GABARAP, modulating autophagic pathways to prevent abnormal protein aggregation. By targeting and stabilizing autophagic flux, these inhibitors could effectively reduce pathological amyloid β accumulation in AD and prevent excessive α-synuclein aggregation in PD. Thus, the dual utility of LIR-based probes as both sensors and inhibitors highlights their potential as powerful tools in the development of targeted therapeutic strategies for neurodegenerative diseases characterized by disrupted proteostasis.

In addition, these probes have become invaluable in cancer research, where autophagy plays a dual role in both tumor suppression and progression. By using LIR-based probes to investigate ATG8 interactions in cancer cells, researchers can better understand how autophagy supports tumor survival under stress and identify new strategies to inhibit autophagy in cancer therapy.

LIR-based probes have been instrumental in uncovering new ATG8-LIR interactions that were previously challenging to detect due to limitations in traditional methods. They offer high specificity and sensitivity, allowing researchers to track dynamic changes in ATG8-protein interactions or changes in lipidated LC3/GABARAP positive vesicles in real-time in various cellular contexts (Park et al., 2023; Lee et al., 2017). Furthermore, LIR-based probes have opened new avenues for studying non-canonical autophagy processes CASM. By detecting ATG8-LIR interactions in these non-canonical contexts, these probes have helped reveal the involvement of ATG8 proteins in immune regulation, membrane repair, and pathogen clearance. In particular, LIR-based probes have provided key evidence supporting the notion that ATG8 proteins are not limited to autophagosome formation but are also integral to processes such as phagosome maturation and lysosomal degradation.

The introduction of LIR-based probes marks a significant advancement in autophagy research, but the potential of these tools has not been fully realized. Future applications may include the development of probes that target specific subsets of ATG8-family proteins or that are tailored to visualize ATG8 interactions or lipidated ATG8-positive vesicles in specific disease models. By refining these tools, researchers will be able to further dissect the molecular mechanisms underlying ATG8’s diverse functions in both autophagy and non-canonical pathways, offering promising new approaches to understanding and treating autophagy-related diseases.

## Irreversible LC3/GABARAP deconjugases

LC3/GABARAP deconjugases are specialized enzymes that precisely remove lipidated ATG8-family proteins from cellular membranes, playing a critical role in the dynamic regulation of ATG8 lipidation and delipidation (Park et al., 2023; Lee et al., 2017) This regulatory mechanism is crucial for both canonical autophagy and non-canonical pathways, allowing researchers to dissect the specific roles of membrane-anchored ATG8 proteins in diverse biological contexts, including pathological states like cancer, neurodegeneration, and immune disorders.

The lipidation process, essential for attaching LC3 and GABARAP proteins to autophagosomal and other cellular membranes, facilitates autophagic functions and can be reversed by ATG8-specific deconjugases, such as ATG4 proteases, which cleave ATG8-family proteins at their C-terminal glycine, recycling them for subsequent autophagy cycles. Interestingly, *Legionella pneumophila* secretes RavZ, a bacterial protein that disrupts host autophagy by irreversibly deconjugating LC3/GABARAP-PE on autophagosomes, mimicking ATG4B in its mechanism (Choy et al., 2012; Yang et al., 2020). RavZ possesses a catalytic domain, several LIRs, and a membrane-targeting (MT) domain that enables dual targeting of autophagic membranes through PI3P binding and mATG8 interactions (Horenkamp et al., 2015; Park et al., 2019a). The positioning of the LIR is crucial for function, with RavZ’s N-terminal LIR facilitating delipidation and ATG4B’s C-terminal LIR enabling LC3B-PE cleavage (Jang et al., 2024; Yang et al., 2020).

Utilizing RavZ as a model, researchers have engineered LC3/GABARAP deconjugases by modifying RavZ’s MT domain and incorporating selective LIRs, resulting in deconjugases specific for distinct ATG8 proteins (e.g., deconjugase-Fy for LC3A/B, deconjugase-St for GABARAP, and deconjugase-TP(T) for LC3C) (Figure 3A) (Park et al., 2023). These engineered deconjugases have shown that GABARAP proteins are essential for aggrephagy, particularly in the degradation of pathological TDP-25 aggregates in neurodegenerative diseases such as ALS (Park et al., 2023).

Manipulating ATG8 lipidation states through deconjugases has significantly advanced our understanding of membrane-anchored ATG8 in various diseases. In neurodegenerative diseases like Parkinson’s and Alzheimer’s, aberrant autophagic flux and disrupted ATG8 regulation contribute to the accumulation of damaged proteins and organelles. Reversing ATG8 lipidation using deconjugases allows researchers to examine how restoring autophagy may reduce pathological protein aggregates, potentially slowing disease progression. In cancer, modulating LC3/GABARAP lipidation sheds light on how autophagy aids tumor cell survival under stress conditions, such as nutrient scarcity or chemotherapy.

Beyond canonical autophagy, LC3/GABARAP deconjugases are essential for understanding non-canonical processes CASM, where ATG8-family proteins are conjugated to single membranes. For instance, in LAP, lipidation of ATG8 proteins is required for the maturation of phagosomes and the clearance of apoptotic cells and pathogens. By regulating the extent of ATG8 lipidation on these phagosomes, deconjugases help control the degradation process and prevent excessive immune activation, which is crucial for preventing autoimmune disorders. In the context of membrane repair, deconjugases also play an important role by removing lipidated ATG8 from damaged membranes once the repair process is complete. This prevents prolonged recruitment of repair machinery, ensuring timely resolution of membrane damage and maintaining cellular integrity.

The ability to modulate ATG8 lipidation through LC3/GABARAP deconjugases offers exciting therapeutic possibilities. Targeting these enzymes could enable the fine-tuning of autophagy in diseases where either excessive or deficient autophagic activity contributes to pathology. In cancer, for instance, inhibiting deconjugases may serve as a strategy to block autophagy-mediated resistance to therapies, thereby enhancing the effectiveness of existing treatments. Conversely, in neurodegenerative diseases, activating deconjugases might help clear toxic aggregates and restore cellular homeostasis.

## Perspective: expanding the understanding of ATG8 lipidation and non-canonical autophagy mechanisms

ATG8-family proteins, such as LC3 and GABARAP, have long been recognized for their roles in canonical autophagy, but their involvement in non-canonical pathways CASM, introduces a new layer of complexity. These processes involve the selective conjugation of ATG8 to single membranes, playing critical roles in immune regulation, membrane repair, and pathogen clearance. The mechanistic details governing these pathways, however, remain elusive, raising several important questions regarding the regulation of ATG8 lipidation and its implications for cellular homeostasis and disease.

One of the most intriguing questions in non-canonical autophagy is what governs the selective conjugation of ATG8 proteins to PS versus PE in pathways like CASM. In canonical autophagy, ATG8 is predominantly lipidated to PE, facilitating the formation and expansion of double-membrane autophagosomes. However, in CASM, ATG8 proteins are conjugated to PS on single membranes, which may serve as a distinct molecular signature for non-canonical autophagy. The mechanisms underlying this lipid specificity are still not fully understood, but it is possible that lipid composition, membrane curvature, and the recruitment of specific ATG machinery play pivotal roles. Additionally, recent findings have highlighted the dual role of ATG5 as a critical regulator in maintaining lysosomal homeostasis by balancing repair and exocytosis, effectively bridging canonical and non-canonical autophagy pathways (Wang et al., 2023). In response to lysosomal damage, ATG5 contributes to lysosomal repair through the CASM pathway by promoting ATG8 conjugation and facilitating interactions with the ESCRT machinery. Simultaneously, under conditions of severe lysosomal damage, ATG5 mediates lysosomal exocytosis, expelling damaged lysosomal contents to alleviate intracellular stress and prevent inflammation. Intriguingly, the loss of ATG5, an E3 ligase essential for Atg8ylation in non-canonical autophagy, not only impairs lysosomal repair but also drives lysosomal exocytosis, revealing its regulatory function in this process. This dual functionality underscores ATG5’s role as a molecular switch that dictates cellular responses to lysosomal damage, balancing repair and exocytosis to maintain homeostasis. Beyond lysosomal maintenance, ATG5’s involvement in Atg8ylation further emphasizes its versatile role in linking canonical and non-canonical autophagy pathways (Wang et al., 2023). These insights into ATG5’s multifaceted functions could pave the way for novel therapeutic strategies targeting lysosomal dysfunction, including lysosomal storage disorders and chronic inflammatory diseases. Understanding how this lipid specificity influences cellular processes, such as immune responses and membrane repair, will be crucial in uncovering how ATG8 lipidation dictates cellular outcomes. Future research may focus on identifying the molecular players that regulate this conjugation selectivity and how disruptions in this process contribute to disease pathology.

The recent development of LIR-based probes has opened up new avenues for studying ATG8-LIR interactions with unprecedented precision. These probes are designed to detect or monitor interactions between ATG8 proteins and their binding partners, which contain LIRs. The application of LIR-based probes could be particularly valuable in disease contexts such as cancer and neurodegenerative disorders, where dysregulated autophagy plays a significant role (Figure 3B). In cancer, for instance, tumor cells often exploit autophagy to survive under stress conditions. By utilizing LIR-based probes, researchers could identify new ATG8 interaction partners involved in tumor progression or resistance to therapies, revealing potential targets for autophagy inhibition. Similarly, in neurodegenerative diseases like Parkinson’s and Alzheimer’s, defective autophagy leads to the accumulation of toxic protein aggregates. LIR-based probes may help uncover novel ATG8 interactions with aggregates or damaged organelles, providing insights into how selective autophagy could be restored in these disease models.

The discovery of LC3/GABARAP deconjugases, which specifically remove lipidated ATG8 proteins from membranes, has opened a new frontier for therapeutic interventions. These deconjugases play a crucial role in regulating autophagic flux by ensuring that ATG8 proteins are properly recycled after autophagosome formation and lysosomal fusion. In diseases where autophagy is either overactive (such as in cancer) or deficient (as in neurodegenerative diseases), manipulating the activity of these deconjugases offers a promising therapeutic strategy. In cancer, inhibiting deconjugases could block autophagy-mediated resistance to therapies, potentially sensitizing tumor cells to chemotherapy or radiation. Conversely, activating deconjugases in neurodegenerative diseases might enhance the clearance of damaged proteins and organelles, alleviating the cellular dysfunction that characterizes these conditions. Future research should focus on developing small molecule inhibitors or activators of these deconjugases, with the goal of fine-tuning ATG8 lipidation in specific disease contexts.

While tools like LIR-based probes and LC3/GABARAP-PE deconjugase have provided new insights, they are still evolving in terms of sensitivity and specificity for tracking real-time dynamics of ATG8ylation in live cells.

## Conclusion

The exploration of ATG8 lipidation in both canonical and non-canonical autophagy processes is a rapidly evolving field, with significant implications for our understanding of cellular homeostasis and disease mechanisms. The questions raised by the selective conjugation of ATG8 to PS versus PE, the potential of LIR-based probes in discovering novel ATG8 interactions, and the therapeutic possibilities offered by targeting LC3/GABARAP deconjugases highlight the importance of continuing research in this area. As our understanding of these processes deepens, the ability to manipulate ATG8 functions will undoubtedly offer new strategies for treating a wide range of disease.
